# Less Is More: Management of Left Atrial Appendage Perforation With Impending Cardiac Tamponade Following Watchman Device Placement

**DOI:** 10.1155/cric/2574323

**Published:** 2025-04-11

**Authors:** Syeda Beenish Bareeqa, Ahmad Nawa, Arooge Towheed, Juwairiya Shuroog, Karthik Ramireddy, Shahabuddin Soherwardi

**Affiliations:** ^1^Tidalhealth Peninsula Regional, Salisbury, Maryland, USA; ^2^Department of Cardiology, Tidalhealth Peninsula Regional, Salisbury, Maryland, USA; ^3^Department of Internal Medicine, Tidalhealth Peninsula Regional, Salisbury, Maryland, USA

**Keywords:** atrial fibrillation, complication, left atrial appendage occlusion, pericardial effusion, stroke

## Abstract

By 2030, the United States will have over 12 million people with atrial fibrillation, which carries a five-fold increase in risk of stroke. Watchman device is an alternative in patients who are poor candidates for anticoagulation. Here, we present a rare case of Watchman device implantation related to left atrial appendage (LAA) perforation noted intraoperatively with portion of the device in the pericardial space. A 93-year-old female with high fall risk and on Coumadin presented for Watchman device placement. During implantation, LAA perforation was noted with exudation of contract in the pericardium during deployment, and decision to deploy the device was made, hoping it would help seal the leak. Retrieving the device was thought to put the patient at risk of bigger perforation. Following which successful pericardial window with temporary drain placement avoided sternotomy and overall had a good outcome. A Watchman device with self-expanding frame covering the left atrial facing surface was deployed, after which a rare but potential complication of perforation was noted during delivery. The device left in place sealed the leak and prevented potential worsening incase device was retrieved. Eventually, this decision improved the outcome of the patient.

**Summary**

• Watchman device has a low periprocedural complication rate; however, complication of LAA perforation needs to be identified promptly.
• Deployment of the device instead of retrieval can lead to an improved outcome of the patient after perforation is noted.

## 1. Introduction

Most serious clinical consequence of atrial fibrillation (AF) is embolic stroke in elderly patients. AF is associated with fivefold increased risk of ischemic stroke and twofold increased risk of mortality [1]. Left atrial appendage (LAA) thrombus is the primary cause of stroke in patients with nonvalvular AF. Watchman device LAA closure/occlusion (LAAO) is a viable alternative for patients with nonvalvular AF who are poor candidates for oral anticoagulant therapy [2]. Long-term outcome of Watchman device was assessed in PREVAIL, PROTECT AF, and PRAGUE-17 trials. PREVAIL trial enrolled 269 patients in intervention group and followed over 18 months. Similarly, PROTECT AF trial assessed 463 patients after Watchman device with mean 2.3 ± 1.1 years follow-up. All three noninferiority trials suggested that LAAO is as effective as direct oral anticoagulation (DOAC). As compared to PROTECT AF trial, PREVAIL trial had lesser device related adverse events. A meta-analysis suggested that there is no significant difference between LAAO and DOAC in all the stroke or systemic emboli. However, there is a marked reduction in hemorrhagic stroke (RR = 0.22) and a significant reduction in cardiovascular and all-cause mortality in LAAO group [3]. These trials suggest that Watchman device is as efficacious as DOACs. Procedural complication of Watchman device is mainly related to transseptal puncture and device implantation including air embolism, pericardial effusion (PE), and tamponade, as well as device embolization [4]. We are reporting a case of puckered Watchman device in the pericardium with high risk of perforation managed with an unconventional clinical approach.

## 2. Case Report

The patient is 93 years old female with the past medical history of COPD, congestive heart failure (CHF), mitral regurgitation, tricuspid regurgitation, and aortic stenosis and nonvalvular AF on warfarin. Despite multiple attempts for correction, the patient continued to have labile INRs (1.3-6.6) with a CHA2DS2-VASc score of 5; history of CHF, age, and female sex; and HAS-BLED of 2. Due to challenges afforded by labile INR, risk for fall in elderly patient and subsequent high risk of bleeding, as well as the patient's preference to come off anticoagulants due to transportation hassle to Coumadin clinic, a Watchman device placement was recommended by multidisciplinary approach.

**Figure 1:**
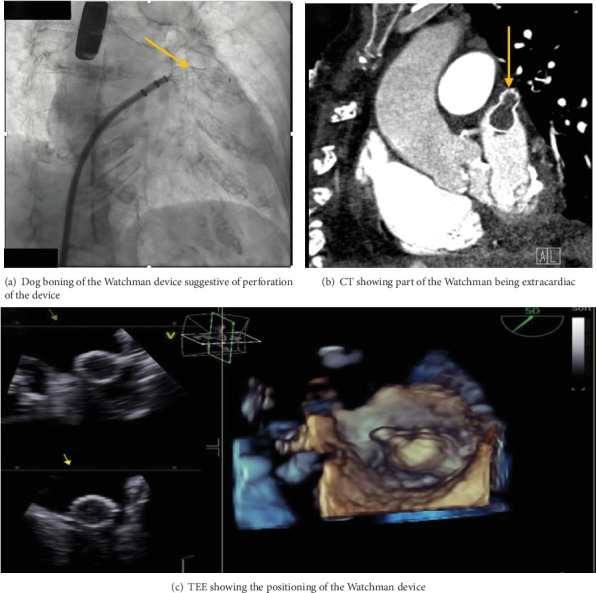
Watchman device placement with impending tamponade.

**Figure 2:**
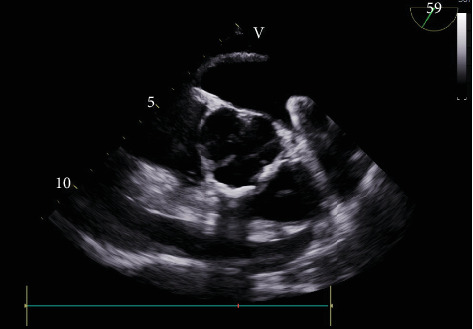
TEE during procedure showing pericardial effusion.

The procedure was performed under general anesthesia, and transseptal access was performed with transesophageal echocardiography (TEE) and fluoroscopy guidance. The LAA ostial measurement was about 22–24 mm in size; therefore, a decision was made by the interventional cardiologist and electrophysiologist to use a 31-mm Watchman flex device. After the transseptal puncture, heparin was administered to maintain an activated coagulation time (ACT) of 250 s throughout the procedure. The pigtail catheter was advanced in the LAA, where contrast injection of the appendage was performed which revealed a bilobed structure of the LAA. The access sheet was advanced to the proximal portion of the LAA, and a flex ball was advanced to the distal LAA. A 31 mm (about 1.22 in.) Watchman flex device was advanced into the left appendage. Owing to the difficult left atrial anatomy, several repositioning and capturing of the device were required. Details of Watchman device positioning can be seen in Figure 1. The device was noticed to be puckered and would not regain its original shape raising concerns for perforation. Contrast angiography demonstrated leakage of the dye into the pericardial space, and TEE confirmed the LAA perforation with impending tamponade. Subsequently, pericardiocentesis was performed through subxiphoid access, and 700 mL of blood was evacuated (Figure 2). Due to the high clot burden, the cardiovascular surgery team was consulted and placed a pericardial window. The patient received two units for packed red blood cell transfusion. Preoperative hemoglobin of 12.2 g/dL, WBC of 6100 *μ*L, creatinine of 0.75 mg/dL remained stable postoperatively (hemoglobin of 11.2 g/dL, WBC of 21,600 *μ*L, which trended down to 9300 *μ*L on Day 1, and creatinine of 0.64 mg/dL). The patient was hemodynamically stable upon transfer to cardiovascular ICU, and then, a day later, the patient was discharged home in stable condition. Postprocedural TEE on Day 2 post-op showed no PE and no evidence of device thrombosis. TTE repeated after 6 months also showed no effusion. The patient was seen in the clinic within a couple of days after discharge for shortness of breath. She was found to be back in AF. She received Lovenox and underwent TEE-guided cardioversion to achieve sinus rhythm. Therapeutic anticoagulation resulted in a drop in 2 g hemoglobin. In the review of TEE images with some reaccumulation of pericardial fluid, the decision was made to discontinue warfarin indefinitely. She continues to do well on low-dose oral aspirin therapy.

## 3. Discussion

PE stands out as the primary serious complication linked to transcatheter LAAO via the Watchman device. According to the NCDR LAA occlusion data registry which consisted of 65,355 patients, it was observed that between 2016 and 2019, the incidence rate of PE was 1.35% (881 patients) [5]. Other studies also suggest that PE occurrence varies between 0.29% and 4.8%, which, although infrequent, holds significant importance given that LAAO is sometimes considered for individuals who may not exhibit clinical symptoms of AF otherwise. Over time, the incidence of PE has diminished, attributed to a rise in implantation numbers and enhanced operator proficiency in LAAO procedures [6–8]. Subsequent trials have demonstrated a decline in PE rates to more acceptable levels, with the most recent PINNACLE FLX trial reporting a complete absence of occurrences [9].

Several observational studies have sought to analyze predictors and uncover the underlying mechanisms behind PE following left atrial appendage closure (LAAC). Previous research has identified various factors, including female gender, paroxysmal AF, alterations in sinus rhythm, device retrieval times, and intraoperative duration, as being positively associated with PE during the perioperative period. Considering that percutaneous LAAC is primarily recommended for individuals with nonvalvular AF and a heightened risk of bleeding from anticoagulant therapy, these underlying conditions may increase vulnerability to procedural complications, with PE being the most prevalent [10].

Anatomical considerations also play a significant role in understanding the elevated risk of PE post-LAAC. The atrial appendage, characterized by its thin-walled structure, is largely covered by the epicardium. Any manipulation of the LAA using a delivery system or closure device could lead to pericardial bleeding due to local trauma, potentially precipitating cardiac tamponade. Additionally, nearby anatomical structures like the pulmonary veins may suffer damage during LAAC, contributing to the incidence of PE. In case of Watchman failure, alternative surgical options include surgical excision or exclusion which includes ligation, stapling, epicardial suturing, or clipping the LAA orifice with devices like AtrialClip [11]. Another option would include replacing the Watchman device with a different size. However, in our case, replacing the device was not an option due to impending tamponade.

In a specific case, our patient encountered complications during the procedure, including LAA perforation and impending tamponade. Despite the challenges, the decision was made to retain the device in place rather than retrieve it—a choice that ultimately yielded a more favorable outcome for our patient.

## 4. Conclusion

LAA occlusion with Watchman device has low periprocedural complication rate; however, LAA perforation needs to be promptly identified. It has been noted that deployment of the device instead of its retrieval is associated with a better outcome. Large scale studies are recommended to validate this conclusion.

## Data Availability

Data sharing is not applicable to this article as no new data were created or analyzed in this study.
